# PB1-F2 Finder: scanning influenza sequences for PB1-F2 encoding RNA segments

**DOI:** 10.1186/1471-2105-12-S13-S6

**Published:** 2011-11-30

**Authors:** David S DeLuca, Derin B Keskin, Guang Lan Zhang, Ellis L Reinherz, Vladimir Brusic

**Affiliations:** 1 Cancer Vaccine Center, Dana-Farber Cancer Institute, 77 Avenue Louis Pasteur, Boston, MA 02115, USA; 2 Broad Institute, 301 Binney Street, Cambridge, MA 02142, USA

## Abstract

**Background:**

PB1-F2 is a major virulence factor of influenza A. This protein is a product of an alternative reading frame in the PB1-encoding RNA segment 2. Its presence of is dictated by the presence or absence of premature stop codons. This virulence factor is present in every influenza pandemic and major epidemic of the 20th century. Absence of PB1-F2 is associated with mild disease, such as the 2009 H1N1 (“swine flu”).

**Results:**

The analysis of 8608 segment 2 sequences showed that only 8.5% have been annotated for the presence of PB1-F2. Our analysis indicates that 75% of segment 2 sequences are likely to encode PB1-F2. Two major populations of PB1-F2 are of lengths 90 and 57 while minor populations include lengths 52, 63, 79, 81, 87, and 101. Additional possible populations include the lengths of 59, 69, 81, 95, and 106. Previously described sequences include only lengths 57, 87, and 90. We observed substantial variation in PB1-F2 sequences where certain variants show up to 35% difference to well-defined reference sequences. Therefore this dataset indicates that there are many more variants that need to be functionally characterized.

**Conclusions:**

Our web-accessible tool PB1-F2 Finder enables scanning of influenza sequences for potential PB1-F2 protein products. It provides an initial screen and annotation of PB1-F2 products. It is accessible at http://cvc.dfci.harvard.edu/pb1-f2.

## Background

The influenza A virus causes epidemics and pandemics [1] in humans, other mammals and birds. The disease severity varies significantly. Recognizing danger factors by sequence analysis helps assess the virulence potential of a given strain. The PB1-F2 is a key danger factor which distinguishes the major flu pandemics of the 20th century (1918 Spanish, 1957 Asian, and 1968 Hong Kong) from the milder 2009 H1N1 “swine flu” pandemic. The presence of this alternative reading frame protein product helps assess virulence of emerging viruses.

PB1-F2 is encoded in an alternative reading frame of the PB1 gene (segment 2). PB1 is an obligatory protein product that enables viral replication, while PB1-F2 is present in some, but not all PB1-coding sequences. It is a pro-apoptotic factor, increasing virulence and the risk of secondary infections [2-4]. PB1-F2 expression is blocked in some strains due to premature stop codons [5]. PB1-F2 increases virulence through the induction of cell death, induction of inflammation, or polymerase activity enhancement [6]. The 2009 H1N1 is characterized by low virulence. It lacks PB1-F2 due to premature stop codons. For example, a stop codon (TAA) at the nucleotide position 128 of PB1 gene in A/Massachusetts/06/2009(H1N1) truncates the potential PB1-F2 after 11 amino acids. In contrast, the virulent A/Brevig Mission/1/1918(H1N1) strain contains codon TCA at position 128, encoding a serine at position 12 of PB1-F2. In the PB1 reading frame, C129A is a silent mutation: both alternative codons, GTC and GTA, encode valine at amino acid position 43 of the PB1 protein. Variants of PB1-F2 have been reported of lengths 57, 87, or 90 amino acids [5].

Since the influenza virus is an RNA virus, the identification of PB1-F2 from heterogeneous sources (PCR products, complete genomes, segments, coding sequences, and fragments) requires translation of the RNA sequence in 3 reading frames. Candidate protein products (hereafter, CPPs) are encoded by all potential open reading frames. All of the potential PB1-F2 products are then identified through their similarity between CPPs and known PB1-F2 sequences. This method was used for annotation of 64 verified entries in Swiss-Prot (with known PB1 proteins) while two entries have protein level evidence.

We present an algorithm and web server for scanning nucleotide sequences for the presence of functional PB1-F2. This web tool helps researchers identify whether a newly isolated sequences en-codes a potential PB1-F2 product.

## Implementation

### Data sources

Sequences were obtained from the Influenza Virus Resource, hosted at NCBI [7]. The nucleotide sequences were extracted from the influenza.fna file provided on their FTP repository in the FASTA format. A set of PB1-F2 sequences was obtained from UniProt to serve as a reference panel. Only reviewed entries from the curated Swiss-Prot entries were used, resulting in 64 panel sequences. Of these 11 (17%), 2 (3%), and 51 (80%) were of lengths 57, 87, and 90, respectively. They represent strains isolated from humans (28 or 44%), birds (32 or 50%), swine (1 or 1.5%) and laboratory strains (3 or 4.5%).

### PB1-F2 identification

CPPs were generated by translating the PB1-encoding influenza A segment 2 nucleotide sequences in 3 reading frames. CPPs shorter than 50 amino acids were considered negative because PB1-F2 proteins of these lengths have been neither described nor hypothesized to exist. To identify whether a given CPP represents PB1-F2, a distance measure was calculated between the query sequences and all panel members. The distance measure was defined as the number of mismatches in a Smith-Waterman alignment as a percentage of sequence length. If the distance to the closest panel member is below a set threshold, the CPP is determined to be a PB1-F2 product, thus distinguishing it from non-PB1-F2 CPPs. The algorithm uses a threshold of 40 to identify PB1-F2 products, which was determined to be discriminatory (see Results).

### Web implementation

All algorithms for this analysis were implemented in the java programming language. The web interface is hosted on an Apache Tomcat server, written in Java and Java Server Pages.

## Results and discussion

Of the 97,260 influenza sequence entries found in the *influenza.fna* source file as of December 2009, 8608 were PB1 (segment 2) entries. After translating all 3 reading frames, and selecting sequences that begin with methionine, and end with a stop codon, 14,637 candidate protein segments (CPPs) were produced with a length between 52 and 398 amino acids.

**Figure 1:**
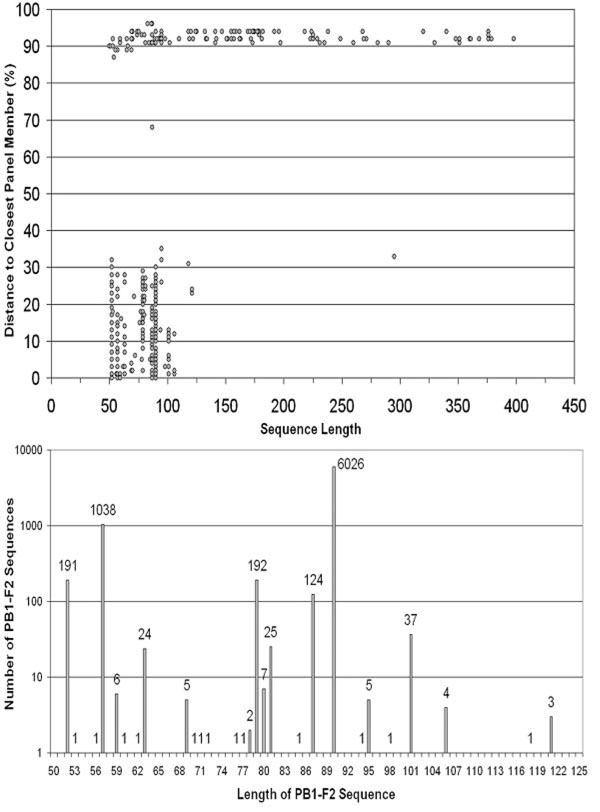
**Summary of PB1-F2 sequences classified by their lengths.** Upper: candidate PB1-F2 peptides show up to 35% sequence dissimilarity compared to a panel of reference sequences from Swiss-Prot. The population with distances above 86% represents non-PB1-F2 ORF translations. Lower: number of PB1-F2 sequences in each group length.

To evaluate the distance measure selectivity across the population of CPPs, the distances were plotted against the sequence length (Figure 1). This revealed 2 major populations: (1) sequences which show less than 36% difference compared to the closest panel member, representing PB1-F2 CPPs and (2) sequences with greater than 86% difference, representing non-PB1-F2 CPPs (e.g. from other reading frames). This figure shows that a conservative cutoff of 40% is effective in distinguishing between these populations.

Figure 1 reveals two prominent outliers, which are caused by unique PB1-F2 open reading frames. The CPP of length 87 and distance 68 is encoded by “defective interfering” influenza virus A/WSN (H1N1) [8]. The second outlier is a CPP from A/shorebird/Korea/S331/2006(H10N9), of length 295 with distance 33. This sequence has a single nucleotide insertion at position 266 in the coding transcript, which causes a frame shift mutation rendering PB1 nonfunctional [9]. These sequences are therefore nonfunctional outliers.

By employing this strategy for PB1-F2 identification, we located PB1-F2-encoding nucleotide sequences throughout the database. Of 8608 PB1-F2 entries, 6417 were determined to encode PB1-F2 (75%). There were 731 sequences in the database that were previously annotated as encoding PB1-F2. Therefore, there we have newly annotated 5686 PB1 entries for the presence of PB1-F2-encoding sequences.

Conservation analysis of PB1 sequences showed that peptides _15_PB1_51_ and _114_PB1_148_ are 94-100% conserved [10]. Since the PB1-F2 start and stop codons fall within these regions respectively, the high level of conservation results in a limited number of PB1-F2 length variants. This is clearly shown in Figure 1. Two major populations of PB1-F2 are of lengths 90 and 57. Several minor populations include lengths 52, 63, 79, 81, 87, and 101. Additional possible populations include the lengths of 59, 69, 81, 95, and 106. Previously described sequences include only lengths 57, 87, and 90. However, we have observed substantial variation in PB1-F2 sequences where certain variants show up to 35% difference to well-defined reference sequences. Therefore this dataset indicates that there are many more variants that need to be functionally characterized. The inspection of multiple sequence alignments has shown the existence of distinct populations that appear to represent various fitness variants. For example, the 206 PB1-F2 sequences that are 52 AA long form 5 major sequence subgroups while 46 sequences that are 101 AA long form three major subgroups. More numerous length variants (e.g. those that are 57 or 90 AA long) have a larger number of major subgroups. Our analysis shows that, unfortunately, characterization of this important virulence factor is neglected by mainstream influenza A sequence characterization. Our web tool is a contribution to this effort since it provides an initial screen and annotation of PB1-F2 products and enables further detailed analysis.

## Conclusion

We have implemented the PB1-F2 analysis algorithm and strategy as a web tool, PB1-F2 Finder. The user can input a nucleotide sequence (either in DNA or RNA format) with or without a FASTA header line. The application then translates the sequence in three reading frames, digests protein products beginning with methionine, and ending with a stop codon, and assesses the presence of PB1-F2 based upon the distance measure described in Methods. Each of these steps is outlined in a full report to the user. The PB1-F2 Finder tool is a contribution to the global fight against pandemic influenza.

## Competing interests

The authors declare that they have no competing interests.

## Authors' contributions

Conception and design: DSD, DBK, ELR, and VB. Acquisition of data: GLZ and DSD. Analysis and interpretation: DSD and GLZ. All authors read and approved the final manuscript.
